# Correlates of Physical Activity among Young Children with Moderate Acute Malnutrition

**DOI:** 10.1016/j.jpeds.2016.10.073

**Published:** 2017-02

**Authors:** Charles W. Yaméogo, Bernardette Cichon, Christian Fabiansen, Ann-Sophie Iuel-Brockdorf, Susan Shepherd, Suzanne Filteau, Alfred S. Traoré, Vibeke B. Christensen, Kim F. Michaelsen, Soren Brage, Henrik Friis, Daniel Faurholt-Jepsen

**Affiliations:** 1 Centre de Recherche en Sciences Biologiques, Alimentaires et Nutritionnelles, Université de Ouagadougou, Burkina Faso; 2 Department of Nutrition, Exercise and Sports, University of Copenhagen, Frederiksberg C, Denmark; 3 Institut de Recherche en Sciences de la Santé, Ministère des Enseignements Supérieurs, de la Recherche Scientifique et de l'Innovation, Ouagadougou, Burkina Faso; 4 Alliance for International Medical Action, Dakar, Sénégal; 5 London School of Hygiene and Tropical Medicine, London, England, United Kingdom; 6 Médecins Sans Frontières, Copenhagen, Denmark; 7 Medical Research Council Epidemiology Unit, Institute of Metabolic Science, Cambridge, England, United Kingdom; 8 Department of Infectious Diseases, Rigshospitalet, Copenhagen, Denmark

**Keywords:** moderate acute malnutrition, young children, physical activity, Africa, AGP, α_1_-acid glycoprotein, CRP, C-reactive protein, HAZ, Height-for-age z score, MAM, Moderate acute malnutrition, MUAC, Midupper-arm circumference, WHO, World Health Organization, WHZ, Weight-for-age z score, SAM, Severe acute malnutrition

## Abstract

**Objective:**

To assess the levels of physical activity among young children with moderate acute malnutrition and to identify clinical, biochemical, anthropometric, and sociodemographic correlates of physical activity.

**Study design:**

In a cross-sectional study, 1609 children aged 6-23 months wore a triaxial accelerometer (ActiGraph GT3x+; ActiGraph, Pensacola, Florida) for 6 consecutive days, from which total physical activity were determined. Data on morbidity were collected based by history and physical examination, and serum C-reactive protein and α_1_-acid glycoprotein were measured.

**Results:**

A total of 1544 (96%) children had physical activity measured, of whom 1498 (97%) completed 6 consecutive days of physical activity recording with a daily median wear time of 24 hours. The mean (±SD) total physical activity was 707 (±180) vector magnitude counts per minute (cpm). Age was negatively correlated with physical activity; compared with children below 12 months of age, those 12-17 months of age, and 18-23 months of age had 51 (95% CI, 26; 75) and 106 (95% CI, 71; 141) cpm lower physical activity, respectively. Fever and malaria were associated with 49 (95% CI, 27; 70) and 44 (95% CI, 27; 61) cpm lower activity, respectively. Elevated serum C-reactive protein and α_1_-acid glycoprotein were both negative correlates of physical activity, and hemoglobin was a positive correlate.

**Conclusions:**

Physical activity declines with age in children with moderate acute malnutrition and is also inversely related to infection and inflammatory status. Future studies are needed to ascertain cause and effect of these associations.

**Trial registration:**

Controlled-Trials.com: ISRCTN42569496.

Physical activity is now well established as important to both the current and future health of children and adolescents.^1^ Higher levels of physical activity in childhood are associated with favorable metabolic and cardiovascular disease risk profiles,^2^ increased well-being, and better cognitive and motor development.^3^, ^4^

Studies using accelerometers have been conducted mainly in well-nourished children.^5^, ^6^, ^7^, ^8^, ^9^ However, little is known about physical activity and their correlates among young children with acute malnutrition, a condition which is likely to affect health and developmental outcomes.^10^ We are aware of 1 study using accelerometers in Ethiopian children admitted to hospital with severe acute malnutrition (SAM),^11^ and studies using questionnaire or direct observation methods for children with moderate wasting in India and Mozambique.^12^, ^13^ No studies investigating physical activity and correlates using accelerometry are available in children with moderate acute malnutrition (MAM), defined as weight-for-height z score (WHZ) between -3 and -2 (World Health Organization [WHO] 2006),^14^ and/or a midupper-arm circumference (MUAC) between 115 and 125 mm.^15^

We aimed to assess the level of accelerometer-based physical activity among 6- to 23-month-old children with MAM in Burkina Faso and to identify clinical, biochemical, anthropometric, and sociodemographic correlates of physical activity.

## Methods

This is a cross-sectional analysis of baseline data from the TreatFOOD study (Controlled-Trials.com: ISRCTN42569496) among 1609 children with MAM. The activity measures were registered as a secondary outcome. The study was conducted in the Province du Passoré, Burkina Faso, at 5 local health centers (Gomponsom, Latoden, Bagaré, Bokin, and Samba) and a nongovernmental organization (Alliance for International Medical Action, Dakar, Senegal). Children were screened by community health workers using MUAC tapes or by designated screening teams with the use of both MUAC and WHZ. Furthermore, children could be referred to a study site from a health center or present at a site on a caretaker's initiative. The final assessment of study inclusion eligibility was performed at site. Children were enrolled if a diagnosis of MAM was confirmed, defined as WHZ between -3 and -2 (WHO 2006)^14^ and/or MUAC between 115 and 125 mm.^15^ In the study site, WHZ was determined using WHO field tables, but anthropometry was later recalculated before analysis. Children were not included if treated for SAM or hospitalized within the past 2 months, were participating in a nutritional program, required hospitalization, or had severe disability.

The study protocol was approved by the Ethics Committee for Health Research in Burkina Faso (2012-8-059), and consultative approval was obtained from the Danish National Committee on Biomedical Research Ethics (1208204). Consent was obtained verbally and in writing (signature or fingerprints) from caretakers of the children before inclusion.

The study was carried out in accordance with the declaration of Helsinki and international ethical guidelines for biomedical research involving human subjects, published by the Council for International Organizations of Medical Sciences. Medical treatment was provided according to an adapted version of the Integrated Management of Childhood Illness guidelines.^16^

### Sociodemographic, Clinical, Biochemical, and Anthropometric Data Collection

At enrollment, a nurse conducted a clinical examination and collected data using structured questionnaires for sociodemographic variables (number of people in the household, house ownership, fuel used in cooking, type of employment, child birth day) and breastfeeding status (breastfed or not on the day of enrollment). Fever was defined as axillary temperature ≥37.5°C. Upper and lower respiratory tract infections were diagnosed by experienced pediatric nurses based on an adapted version of the Integrated Management of Childhood Illness.^16^ The morbidity data presented were collected at enrollment when initiating the activity measurement, and not repeated during the measurement period. Venous blood (2.5 mL) was collected to carry out rapid antigen test for *Plasmodium falciparum* malaria (SD Bioline, Malaria antigen P.f.), and to determine hemoglobin level (HB 301; HemoCue, Ängelholm, Sweden); anemia was defined as <11 g/dL. Serum was separated and stored at -20°C. C-reactive protein (CRP) and α_1_-acid glycoprotein (AGP) were determined using a simple sandwich enzyme-linked immunosorbent assay.^17^ We defined CRP ≥10 mg/L and AGP ≥1 g/L as abnormal, indicating systemic inflammation. Weight (model 881; Seca, Hamburg, Germany) and length (wooden length board) were measured to the nearest 100 g and 1 mm, respectively. MUAC was measured to nearest 1 mm at the midpoint between the olecranon and the acromion process using a standard measuring tape. All measurements were done in duplicate. The anthropometry measurements were done by trained staff and equipment was checked daily. Standardization sessions were carried out prior to the start of the trial to ensure precision and accuracy of measurements. During the trial, anthropometry staff were closely supervised by the anthropometry team leader and the site supervisor. Movement ability of the children was defined as not able to crawl/walk, able to crawl, or able to walk as assessed by measurement staff based on observation using an adapted version of the Malawi Developmental Assessment Tool.^18^

### Physical Activity Measures and Questionnaire Data

Physical activity was measured objectively using a triaxial accelerometer (ActiGraph GT3X+; ActiGraph, Pensacola, Florida). The accelerometer was attached to an elastic belt placed on the skin at the right side of the hip and worn for 6 consecutive days (6 × 24 hours). Caretakers were instructed to only let enrolled children wear the device and to make sure that the accelerometer was placed on the right hip during the monitoring period. Monitors could be removed during bathing. We used data recorded by the device starting 7 hours after leaving the clinic and ending 7 hours before returning to the clinic to avoid recording unusual activity caused by the need to attend the clinical appointments. After monitor removal, the caregiver was interviewed using a structured physical activity questionnaire including perception and acceptability of the device, episodes of device removal, and whether children were carried and if so how many times per day (coded as never, 1-2 times per day, 3-6 times per day, more than one-half of the day, or all the day).

### Data Analyses

The recorded activity data were uploaded from the monitors using the Actilife 6 Software (ActiGraph). Raw accelerometer data were collected at a rate of 100 Hz. Data were integrated to 10-second epochs to permit detection of short bouts of activity.^6^, ^19^ Each axis (x, y, and z) was converted to counts per min (but still in 10-second resolution), following which vector magnitude was calculated as the square root of sum of the three squared count values. We included data from all times of the measurement period including night (and other sleep) time in the analysis, except the 7 hours in the beginning and end of the file (see above) and periods marked as nonwear. We defined nonwear time as continuous runs of zero activity ≥90 minutes. Days with <8 hours valid wear data and participants with <1 valid day of recording were excluded from the present analyses. We calculated total physical activity as mean vector magnitude over valid days (counts per minute, cpm).

All statistical analyses were performed using Stata v 12 (StataCorp, College Station, Texas). Anthropometric WHZ and height-for-age z score (HAZ) were calculated using the package “zscore06” in Stata. Variables were tested for normality by histograms and Shapiro-Wilk tests. Means ± SD were calculated for normally distributed variables and median (IQR) for non-normally distributed variables. To determine associations between activity and covariates, we first built unadjusted models comparing volume of physical activity between groups of morbidity, biochemistry, and anthropometry. Second, we adjusted for age and sex (model 1), and finally for all covariates including age, sex, paternal and maternal profession, season of measurement, breastfeeding, number of children under 5 years of age in household, carrying status, and movement ability (model 2). The Figure is based on random effect mixed models with age and sex as fixed effects and child as a random effect.

## Results

A total of 1609 eligible children, predominantly Mossi, were enrolled in the study from September 2013 to August 2014, after consent from caretakers. Of the 1609 enrolled, 29% of children were enrolled based on MUAC only, 50% based on WHZ and MUAC, and 21% based on WHZ only, as previously reported.^20^ Among these, 1544 (96%) children had baseline physical activity data and were included in the analysis. The median (IQR) age was 11.3 (8.2; 16) months. More than one-half the children were girls, and almost all children were breastfed (Table I). The majority of children were from families with fuel for cooking based on “coal/wood/straw,” and from families who were owners of their own house. The mean (±SD) MUAC, WHZ, and HAZ were 123 (±4) mm, -2.2 (±0.5), and -1.7 (±1.1), respectively. As previously reported,^21^ comorbidities were common **(**Table II**)**. The 65 (4%) children who were excluded from analyses did not differ from those included with respect to age, proportion of girls, proportion of breastfeeding or prevalence of fever, positive malaria test, diarrhea, cough, or raised levels of CRP or AGP (data not shown).

### Level and Associations of Physical Activity

Of the 1544 children with physical activity data, 1498 (97%) completed 6 consecutive days of recording with a daily median wear time of 24 hours. At the first day of enrollment (7 hours excluded), the 25th, 10th, 5th, and 1st percentiles of wear time were 16.7, 11.4, 11.4, and 10.6 hours, respectively. The mean (±SD) total physical activity was 707 (±180) cpm, with age being inversely associated (Table I**)**. Compared with children below 12 months of age, those aged 12-17 months and 18-23 months had 34 (95% CI, 13; 54) and 121 (95% CI, 97; 145) cpm lower activity, respectively. Judging from the diurnal pattern of activity, waking hours began on average between 6 a.m. and 7 a.m., from which time activity increased up to 9 a.m., then declined to reach a local nadir at around 2 p.m. and increased again until reaching a peak at 7 p.m. and decreased thereafter (Figure). The highest accumulation of activity occurred between 6 p.m. and 7 p.m. During daytime hours, younger children were more active than older children. In unadjusted models, children who were not able to crawl/walk had 51 (95% CI, 29; 72) cpm lower activity and 38 (95% CI, 14; 62) cpm higher activity compared with those classified as able to crawl or walk, respectively. There was no difference between boys and girls but children of farming parents had higher activity levels. Ethnic group and socioeconomic status based on fuel for cooking and house ownership were not associated with activity (*P* > .20, data not shown). Breastfed children were 239 (95% CI, 202; 276) cpm more active than those not breastfed. Neither anthropometric indicators nor admission criteria were associated with physical activity (*P* > .20, data not shown).

Multivariable analysis, including all variables from Table I as covariates, did not change the difference between age groups; children younger than <12 months of age had higher adjusted activity than children aged 12-17 months (β 51, 95% CI, 26; 75) and 18-23 months (β 106, 95% CI, 71; 141). The adjustment only marginally affected the role of breastfeeding (β 199, 95% CI, 160; 239) but increased the impact of ability to crawl (β 71, 95% CI, 48; 93) and walk (β 71, 95% CI, 37; 106). Also father's profession and season for measurement were unaffected by adjustment. On the contrary, the impact on activity by mother's profession or carrying status seemed to be confounded and no longer significant after adjustment.

In unadjusted analyses, most measures of clinically assessed morbidity (except diarrhea), anemia, and inflammation (assessed using acute phase proteins) were negative correlates of physical activity (Table II**)**. In the adjusted models, signs of respiratory tract infection (upper and lower respiratory tract infections, cough) were no longer associated with activity. However, fever, malaria, anemia, and inflammation remained strongly associated with lower volume of activity, with estimates only marginally changed compared with unadjusted models.

Finally, none of the anthropometric measures, WHZ, HAZ, and MUAC, were associated with physical activity; HAZ (β -5, 95% CI, -13; 3), WHZ (β 9, 95% CI, -9; 28), and MUAC (β 1, 95% CI, -2; 3). None of the selection criteria was associated with physical activity; WHZ only vs WHZ and MUAC (β 4, 95% CI, -19; 26) or MUAC only vs WHZ and MUAC (β 6, 95% CI, -20; 33).

## Discussion

Among this large group of young (6-23 months of age) children from Burkina Faso with MAM, we found activity to be correlated with measures of sociodemographic status and morbidity.

Few studies from low-income countries are available using similar equipment, but most of these used a different study design in that they did not collect data for the full 24 hours of the day, and there were also differences with respect to accelerometer data reduction methods. This makes direct comparison somewhat difficult but do allow relative comparisons of the within-population associations with covariates. A single-center study from Ethiopia in a small group of children with SAM used an identical approach to the one used in the present study.^11^ Compared with the Ethiopian study, we found a 5-fold higher level of physical activity in children with MAM from Burkina Faso (707 vs 141.5 cpm), suggesting that the degree of malnutrition is a likely determinant of movement in this age group. We did not find any difference in activity between boys and girls among children with MAM in our study, possibly reflecting that activity of young healthy children may not differ by sex. The lack of difference in physical activity between boys and girls is consistent with studies from Belgium among 20-month-old children,^6^ from Australia among 19-month-old children,^22^ from Sweden among 2-year-old children,^8^ and from The Netherlands.^9^ With respect to the association with motor development milestones, ability to crawl or walk were associated with higher activity levels compared with children who are less developed; remarkably, this effect was observed independently of age and how much the child was being carried.

Diurnal patterns in activity showed peaks of activity in the morning and afternoon. This could represent times where children were more engaged in unstructured play. The decrease of activity observed during midday is likely due to feeding and subsequent napping, although we have no observational data to confirm this. These diurnal patterns are, however, consistent with studies among 36-month-old children from New Zealand^23^ and Australia.^24^

Although poor nutritional status is considered to have a negative effect on activity,^25^, ^26^ we did not see any association between the anthropometric measures and physical activity, possibly because all children included in this cohort had anthropometric measures within the narrow MAM range. Breastfed children, who were younger, seemed to be more active. Although this could have been influenced by the children being carried, both the age and the breastfeeding effects remained significant after adjustment for how much the child was carried, suggesting that breastfeeding may play an important role in the nutritional support for activity of children with MAM.

The higher activity seen among children from farming families may have been because they spent more time in the field either playing or in field activities. They could also have been carried while the mother works in field, which could have influenced the registered movement (passive activity); indeed, this effect was no longer significant when other covariates, including carrying status, were being considered. Children enrolled during the rainy season were more active, which was also reported in a study from Zanzibar.^25^ Because farming activity is linked to the rainy season, this could account for the greater physical activity among children measured during this season.

We found a negative association between infection at enrollment and physical activity. Our results were consistent with the study in Zanzibar, where malaria was found to be negatively associated with children's activity, likely because of inflammation, lethargy, and poor appetite.^25^ Children compensate for lack of dietary energy by decreasing energy expenditure through reduced physical activity.^27^ Infection and inflammation can lead to a reduction in body mass, which may reduce capacity to perform work or movement.^28^

Higher hemoglobin was associated with greater physical activity, as has been seen also for children in Mexico and Indonesia.^29^, ^30^ Anemia may be related to iron deficiency or to inflammation. Irrespective of the underlying cause, anemia leads to lower oxygen-carrying capacity or reduced cellular oxidative capacity resulting in low energy production associated with low activity levels. It is notable that anemia remained significantly associated with activity in the multivariable analysis, which included infection indicators, suggesting these other mechanisms also may be important. Anemia may reduce children's endurance as has been found in adolescents and school children.^31^

The strengths of this study include its large sample size, the use of an objective measure of activity with high time resolution, and high compliance covering all 24 hours of the day. Also, this is the first study to investigate physical activity and correlates among young children with MAM using accelerometers. The limitations of the study include a lack of age-matched control data from well-nourished young children from Burkina Faso. Also, most of the children were breastfed and may to some extent have been carried by caregivers and the lack of synchronous activity registration of the caregiver and/or logs did not allow detailed distinguishing passive movement of the child caused by carrying from actual physical activity of the child. Another potential limitation is the lack of a sleep log that would have enabled better comparison with other studies that include only daytime activity.

Physical activity declines with age and is associated with infection and inflammation status in children with MAM. However, because younger children are more likely to be carried, future studies should use both accelerometers and activity logs to improve assessment and aid the distinction between passive and active movement.

## Figures and Tables

**Figure:**
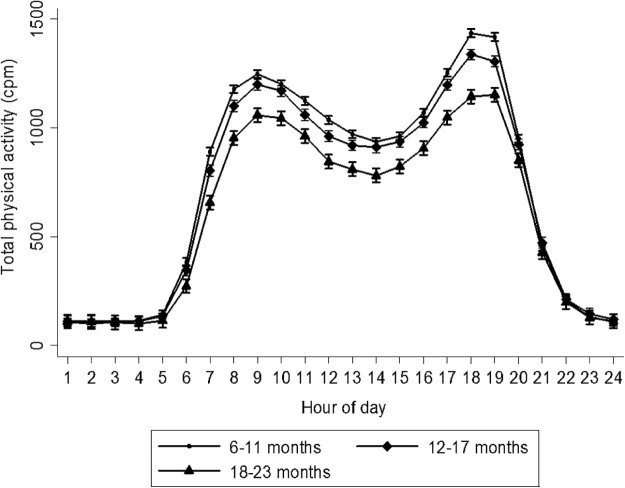
Diurnal patterns in physical activity association with age group. Data represent age- and sex-adjusted means of total physical activity based on random effect mixed model. *cpm*, count per minute.

**Table I:** Background characteristics and physical activity among 1544 children with MAM

		Total physical activity (cpm)				
	n (%)	Mean physical activity (±SD)	Unadjusted β (95% CI)*	*P* value	Adjusted β (95% CI)†	*P* value
Age (mo), median (IQR)	11.3(8.2;16)					
6-11	837(54.2)	738(173)	Ref		ref	
12-17	441(28.6)	705(176)	−34(−54;−13)	.001	−51(−75;−26)	<.001
18-23	266(17.2)	617(179)	−121(−145;−97)	<.001	−106(−141;−71)	<.001
Sex						
Girls	844(54.7)	704(172)	Ref		ref	
Boys	700(45.3)	711(191)	7(−11;25)	.451	10(−7;26)	.273
Mother's profession						
Farmer	1457(94.4)	710(181)	Ref		ref	
Others	87(5.6)	667(165)	−43(−82;−4)	.032	5(−39;50)	.818
Father's profession						
Farmer	1411(91.4)	711(181)	Ref		ref	
Others	133(8.6)	667(160)	−45(−77;−13)	.006	−41(−77;−4)	.031
Number of children <5-y-old in household						
1-2	783(50.8)	705(177)	Ref		ref	
3-4	553(35.8)	716(183)	11(−9;31)	.295	7(−12;25)	.486
≥5	207(13.4)	692(182)	−13(−41;14)	.348	−19(−45;7)	.151
Season of measurement						
Dry season	1015(65.7)	687(173)	Ref		ref	
Rainy season	529(34.3)	745(188)	58(39;77)	<.001	52(34;70)	<.001
Breastfeeding						
Breastfed	1456(94.4)	720(171)	Ref		ref	
Not breastfed	86(5.6)	481(180)	−239(−276;−202)	<.001	−199(−239;−160)	<.001
Carrying status						
Never carried	16(1.1)	575(174)	ref		ref	
1-2 times per d	407(27.1)	682(181)	107(17;196)	.020	34(−50;117)	.427
3-6 times per d	980(65.4)	717(778)	141(53;230)	.002	50(−33;133)	.234
More than one-half of the d	91(6.1)	739(187)	164(68;259)	.001	83(−6;172)	.068
All the d	5(0.3)	644(185)	68(−112;249)	.456	12(−155;178)	.891
Movement ability						
Not able to crawl/walk	403(26)	694(168)	ref		ref	
Crawling	726(47)	744(181)	51(29;72)	<.001	71(48;93)	<.001
Walking	415(27)	656(175)	−38(−62;−14)	<.001	71(37;106)	<.001

*β*, regression coefficient (difference in physical activity volume between groups); *ref*, reference group.

Sample sizes: Breastfeeding (n = 1542), number of children in household (n = 1543).

* Unadjusted linear regression; unadjusted comparisons of volume of physical activity.

† Linear regression-based estimates of total PA mutually adjusted for age, sex, mothers' profession, father's profession, number of children <5-y-old in household, season of measurement, breastfeeding, carrying status, and movement ability.

**Table II:** Associations of volume of physical activity with morbidity, biochemistry among 1544 children with MAM

		Volume of physical activity (cpm)			Adjusted linear models			
					Model 1		Model 2	
	N (%)	Mean (SD)	Δ physical activity (95%CI)	*P* value	β (95% CI)	*P* value	β (95% CI)	*P* value
Clinical examination, presence of:								
Upper respiratory tract infection	228(14.8)	688(184)	−23(−48;2)	.074	−17(−42;8)	.173	−11(−35;15)	.354
Lower respiratory tract infection	361(23.4)	690(189)	−22(−44;−1)	.039	−21(−41;−0.1)	.049	−15(−35;5)	.148
Fever	273(17.7)	678(196)	−36(−59;−12)	.003	−40(−63;−17)	.001	−49(−70;−27)	<.001
Cough	413(26.8)	685(667)	−30(−50;−9)	.004	−27(−46;−7)	.008	−16(−36;3)	.096
Diarrhea	90(5.8)	704(190)	−3(−42;35)	.868	−7(−44;31)	.729	−2(−38;34)	.902
Malaria	615(40)	677(185)	−51(−69;−33)	<.001	−46(−63;−27)	<.001	−44(−61;−27)	<.001
Biochemical data*								
CRP ≥10 (mg/L); (ref: < 10 mg/L)	361(24)	669(189)	−50(−71;−29)	<.001	−47(−68;−26)	<.001	−51(−71;−31)	<.001
AGP ≥1 (g/L); (ref: < 1 g/L)	986(65.7)	679(181)	−79(−98;−61)	<.001	−69(−87;−50)	<.001	−60(−78;−42)	<.001
Hb <11 (g/dL); (ref: Hb ≥11 g/dL)	1087(70.4)	693(184)	−49(−68;−29)	<.001	−44(63;25)	<.001	−37(−56;−19)	<.001

*Δ* physical activity, difference in physical activity between group and reference; *Hb*, hemoglobin.

Data are based on linear regression models: *Δ* physical activity (95% CI) is difference in volume of physical activity compared with reference group, model 1 (adjusted for age and sex), model 2 (adjusted for age, sex, mother's and father's profession, season of measurement, breastfeeding, number of children <5-y-old in household, carrying status, and movement ability).

Sample sizes: Upper respiratory tract infection (n = 1542), lower respiratory tract infection (n = 1543), cough (n = 1541), malaria (n = 1536), and CRP and AGP (n = 1502).

* CRP ≥10 mg/L and AGP ≥1 g/L defined as abnormal indicating systemic inflammation. Anemia defined as hemoglobin <11 g/dL.
